# Social isolation and risk of schizophrenia: a cohort and Mendelian randomization study

**DOI:** 10.1192/j.eurpsy.2025.358

**Published:** 2025-08-26

**Authors:** Q. Wu, Y. Nie, J. Zhang, Y. Y. Liang

**Affiliations:** 1 Center for Sleep and Circadian Medicine, The Affiliated Brain Hospital of Guangzhou Medical University, Guangzhou, China

## Abstract

**Introduction:**

Social isolation refers to the lack of objective social contact and is one of the health risk factors. Previous studies have shown that social isolation is associated with several physical and mental disorders. However, the relationship between social isolation and schizophrenia remains unclear.

**Objectives:**

This study aimed to investigate whether there is a potentially causal association between social isolation and schizophrenia.

**Methods:**

The UK Biobank (UKB) is a large population-based cohort study. In this study, social isolation was assessed using self-reported questionnaires. Participants were categorized into socially isolated and non-isolated groups based on the scores. We identified cases of schizophrenia through hospitalization records and death registrations in the UKB. Propensity score matching was used to reduce the influence of confounding factors. We estimated the hazard ratios (HRs) of social isolation to schizophrenia using Cox regression. In addition, we estimated the causal association between social isolation and schizophrenia by two-sample Mendelian randomization analysis. Genetic data for social isolation and schizophrenia were extracted from the UKB and the Psychiatric Genomics Consortium, respectively.

**Results:**

315 cases of schizophrenia were documented during a mean follow-up of 12.3 years. After adjusting for demographic, socioeconomic, and lifestyle factors, the risk of schizophrenia in the socially isolated group was 1.73 times higher than that in the non-socially isolated group (95% CI, 1.37-2.18). Results from two-sample Mendelian randomization analysis showed that participating in more other group activities was associated with a reduced risk of schizophrenia (OR, 0.49; 95% CI, 0.31-0.77).

**Conclusions:**

Social isolation was potentially causally associated with an increased risk of schizophrenia. The results of this study emphasize the importance of reducing the risk of developing schizophrenia through initiatives to reduce social isolation and increase social activities.

**Disclosure of Interest:**

None Declared

